# Rational construction of free-standing P-doped Fe_2_O_3_ nanowire arrays as highly effective electrocatalyst for overall water splitting

**DOI:** 10.1039/d0ra08586c

**Published:** 2021-01-04

**Authors:** Yong Li Tong, Bao Qian Chi, Dong Li Qi, Weiqiang Zhang

**Affiliations:** School of Science, Shenyang Ligong University Shenyang 110179 P. R. China tyl.tongyongli@163.com; School of Environmental and Chemical Engineering, Shenyang Ligong University Shenyang 110870 China weiqiangln@163.com

## Abstract

Designing electrode structures with high activity is very significant for energy conversion systems. However, single electrode materials often exhibit poor electronic transportation. To address this issue, we prepared P-Fe_2_O_3_ nanowire arrays through a convenient hydrothermal and phosphation method. The as-obtained electrode materials exhibited excellent electrocatalytic performance, which could be attributed to the P element decoration improving the reaction active sites. The as-obtained P-Fe_2_O_3_-0.45 nanowire arrays exhibited excellent OER activity with a low overpotential of 270 mV at 10 mA cm^−2^ (72.1 mV dec^−1^), excellent HER performance with a low overpotential of 126.4 mV at −10 mA cm^−2^, a small Tafel slope of 72.5 mV dec^−1^ and long durability. At the same time, the P-Fe_2_O_3_-0.45 nanowire arrays possessed a low cell voltage of 1.56 V at 10 mA cm^−2^.

## Abstract 2

Designing electrode structures with high activity is very significant for energy conversion systems.

## Graphical abstract

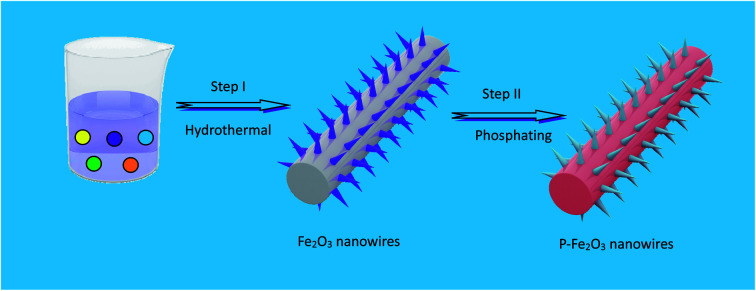

## 1. Introduction

To meet the challenges of energy crisis and develop alternative energy sources for traditional fossil fuels, the development of new energy devices has become very important for future economic and strategic development.^1–6^ Among various energy storage systems to obtain renewable hydrogen production, electrocatalytic water splitting, which contains two half reactions of hydrogen evolution reaction (HER) and oxygen evolution reaction (OER), is probably one of the most achievable methods.^7–10^ However, hydrogen is considered as a clean energy source due to its high energy density, easy purification and environmental friendliness. In previous studies, Pt catalysts have received extensive research attention, and are considered as the most efficient electrocatalysts.^11^ However, it high price limits their further widespread using. In addition, the slow OER kinetic reaction, involving a one-step four-electronic process, often limits the efficiency of overall water splitting.^12,13^ To improve the efficiency of overall water splitting, the development of highly active and inexpensive catalysts is very important to improve the energy conversion efficiency.

Recently, Fe_2_O_3_ has obtained intensive attention due to its low cost and adjustable composition.^14,15^ However, intrinsic poor conductivity restricts its large scale applications. How to improve the electrocatalytic performance of oxides has become a key research focus, and element doping has been ascribed as an efficient method to improve the intrinsic activity of electrocatalysts, which could accelerate the reaction kinetics rates. In addition, transition metal phosphides (TMPs) have been utilized widely for studying overall water splitting due to their high catalytic performance, high stability and high faradic efficiency in all pH ranges. Since P compounds can be captured by acting as protons, while promoting the formation of peroxides intermediate OER.^16–18^ Simultaneously, phosphides can be partially oxidized at the oxidation potential, and these intermediate oxidation states, as seen in oxides and phosphates, all have high OER capabilities. As a consequence, metal phosphides have good electrocatalytic properties. Thus, constructing excellent electrode materials by combining high conductivity materials or element doping has been considered as an effective method for high electrocatalytic performance. On the other hand, the microstructure of the material has a serious impact on the performance of the material. Among the various morphologies, the nanowire arrays obtained a lot of attention. The nanowire arrays show a stable three-dimensional structure, and at the same time, there are a large number of gaps between adjacent nanowires, which is beneficial to charge transfer.^19^ In addition, carbon cloth possesses excellent conductivity, robust skeleton and large surface area, and therefore can effectively increase the contact area between catalysts and electrolytes.

Herein, we fabricated P-Fe_2_O_3_ nanowire arrays through a convenient hydrothermal and phosphation method. The as-obtained P-Fe_2_O_3_-0.45 nanowire arrays exhibited excellent OER activity with a low overpotential of 270 mV at 10 mA cm^−2^, a low Tafel slope of 72.1 mV dec^−1^ and an excellent cycle stability as compared to single Fe_2_O_3_ nanowire arrays. At the same time, the P-Fe_2_O_3_-0.45 nanowire arrays also showed an excellent HER performance, a low cell voltage of 1.56 V at 10 mA cm^−2^ and excellent cycle stability. Thus, we have successfully fabricated novel P-Fe_2_O_3_ nanowire arrays through a simple hydrothermal process.

## 2. Experimental section

All chemical reagents were used as purchased. Ferric nitrate, sodium sulfate, PVP and NaH_2_PO_2_·H_2_O were purchased from Aladdin. Before the experiment, a piece of carbon (4 × 4 cm) was immersed in HNO_3_ solution for 30 min for improving the hydrophilicity of the carbon cloth. Synthesis of Fe_2_O_3_ nanowire arrays: in a typical process, 2 mM ferric nitrate, 2 mM sodium sulfate and 1.0 g PVP (polyvinyl pyrrolidone) were dissolved in 40 ml deionized water. Then, the pretreated carbon cloth and above solution was placed in an autoclave and kept at 100 °C for 10 h. Finally, the as-synthesized Fe_2_O_3_ nanowire arrays were annealed at 350 °C for 2 h in air.

Synthesis of P-Fe_2_O_3_ nanowire arrays: the as-obtained precursors and NaH_2_PO_2_·H_2_O were placed at both ends of a tubular furnace, and NaH_2_PO_2_·H_2_O was at the upstream side. The phosphation process was conducted at 300 °C for 2 h with a heating rate of 2 °C min^−1^ under continuous Ar atmosphere. To further investigate the effect of P doping, different contents of NaH_2_PO_2_·H_2_O (0.25, 0.45 and 0.65 g) were added to fabricate P-Fe_2_O_3_ nanowire arrays. According to the phosphorus content, they were named as P-Fe_2_O_3_-0.25, P-Fe_2_O_3_-0.45 and P-Fe_2_O_3_-0.65, respectively.

### 2.1 Materials characterization

Crystal structure and morphology characteristics were investigated using a Shimadzu XRD-6000 diffractometer, a scanning electron microscope (FESEM, ZEISS Merlin Compact) and a TEM (FEI Tecnai F20). X-ray photoelectron spectroscopy (XPS) measurements (ESCALAB250) with an Al Kα source were used to investigate the phase composition and crystal structure of samples. The surface area of P-Fe_2_O_3_ and Fe_2_O_3_ nanowire arrays was analyzed through N_2_ absorption and desorption isotherms. The pore sizes were measured through the adsorption–desorption isotherms.

### 2.2 Electrocatalytic characterization

Electrocatalytic performance was investigated by conducting tests such as cyclic voltammetry (CV), linear sweep voltammetry (LSV) and *i*–*t* curve measurement using the standard three electrode system in 1.0 M KOH aqueous solution. The as-obtained samples were used as the working electrodes and Hg/HgO was used as the reference electrode. Pt plate was used as the counter electrode for OER (graphite counter electrode for HER). All potentials were calculated using the following equation:

1 *E*_RHE_ = *E*_Hg/HgO_ + 0.098 V + 0.059 V × pH (pH ∼ 13.6)

where *E*_RHE_ is the potential of the reversible hydrogen electrode, *E*_Hg/HgO_ represents the experimentally measured potential against Hg/HgO reference electrode. All overpotentials (*η*) of the OER were calculated through the following equation:

2 *η* = *E*_RHE_ − 1.23

Electrochemical impedance spectra (EIS) were evaluated in the frequency range of 10 to 100 kHz.

## 3. Results and discussion

**Fig. 1:**
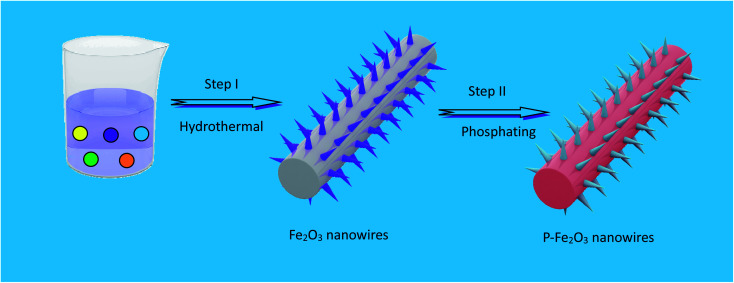
Schematic illustration of the formation procedure of P-Fe_2_O_3_ nanowire arrays on carbon cloth.

The fabrication process of P-Fe_2_O_3_ nanowire arrays is shown in Fig. 1. Firstly, Fe_2_O_3_ nanowire arrays were grown on carbon cloth through a simple hydrothermal method. This was followed by thermal annealing, where FeOOH samples were converted to Fe_2_O_3_ nanowire arrays with a large specific surface area structure. Then, through controllable phosphation with NaH_2_PO_2_ as the P source, the as-prepared P-Fe_2_O_3_ nanowire arrays supported on carbon cloth can be obtained.

**Fig. 2:**
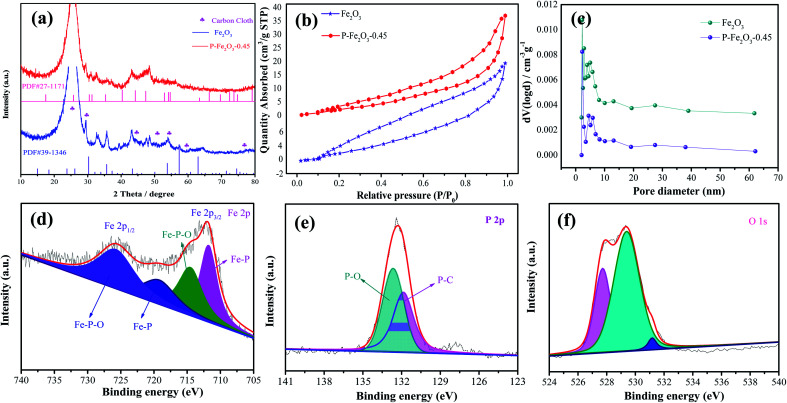
Structure characterization of the as-fabricated P-Fe_2_O_3_ samples (a) XRD patterns (b) BET, (c) pore size distributions, XPS spectrum of the P-Fe_2_O_3_ samples (d) Fe 2p (e) P 2p (f) O 1s.

**Fig. 3:**
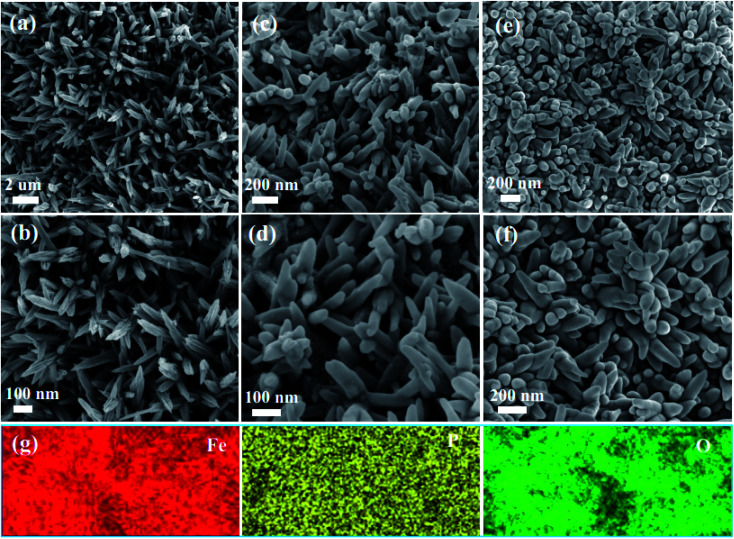
SEM images of the as-obtained Fe_2_O_3_ nanowire arrays at different magnification (a and b) Fe_2_O_3_ nanowire arrays (c and d) the as-obtained P-Fe_2_O_3_-0.25 nanowire arrays (e and f) the as-obtained P-Fe_2_O_3_-0.65 nanowire arrays (g) element mapping.

The crystal structure of the as-obtained samples was investigated through XRD, as shown in Fig. 2a. The diffraction peaks could be identified as Fe_2_O_3_ phase (JCPDS no. 39-1346). The strong diffraction peaks could be indexed to the carbon cloth (JCPDS no. 41-1487). After reacting with the phosphate, a Fe_2_P phase (JCPDS no. 27-1171) could be found. The remaining diffraction peaks were attributed to Fe_2_O_3_. Fig. 2b exhibits the N_2_ isotherms of the as-obtained Fe_2_O_3_ and P-Fe_2_O_3_ nanowire arrays. It was obviously found that P-Fe_2_O_3_ nanowire arrays showed a typical type-IV isotherm with an H3 hysteresis loop at a relative pressure range from 0 to 1.0, indicating that the samples possessed mesoporous characteristic.^19^ From the curves, the BET surface areas of Fe_2_O_3_ and P-Fe_2_O_3_ nanowire arrays were calculated to be 29.562 and 36.353 m^2^ g^−1^, respectively, indicating that P-Fe_2_O_3_ nanowire arrays had a larger surface area than Fe_2_O_3_ nanowire arrays, which benefited in the improvement of the electrocatalytic performance. In addition, the pore distribution curve of the as-prepared samples is shown in Fig. 2c; it was found that the pore diameter of Fe_2_O_3_ and P-Fe_2_O_3_ nanowire arrays were 1.732 and 2.346 nm, respectively. XPS measurement was used to analyze the surface elemental composition of the as-obtained Fe_2_O_3_ and P-Fe_2_O_3_ nanowire arrays. Fig. 2d exhibits the Fe 2p spectrum, which contains four peaks. The binding energy at 711.8 eV can be indexed to Fe 2p_3/2_, (Fig. 3f) can be divided into and 2p_1/2_ peaks. The peaks at 714.6 eV belong to Fe–P–O. The Fe 2p_1/2_ peaks were fitted into the Fe–P (719.8 eV) and Fe–P–O (726.1 eV) bonds.^20,21^ At the same time, it was demonstrated that the Fe–P bond possessed high activity for oxygen reduction. In Fig. 2e, the P 2p spectrum can be divided into two peaks. The binding energy of 131.9 eV corresponds to P–C bonds.^22^ The peaks at 132.8 eV can be attributed to the P–O bonds.^23^Fig. 2f shows the O 1s spectrum, which contains three kinds of oxygen. The binding energies of 527.9 eV, 529.8 eV and 532.5 eV can be ascribed to the metal oxygen bonds, oxygen ions and physicochemical water on the surface of the active material, respectively.^24–26^

The morphology characteristics of the as-obtained samples are shown in Fig. 3. From Fig. 3a, it was found that the as-fabricated Fe_2_O_3_ nanowire arrays were uniformly distributed on the surface of the carbon cloth. Fig. 3b shows the high magnification SEM images. It was obviously found that the surface of Fe_2_O_3_ nanowire arrays was very smooth, and possessed an average width of 50 nm. Fig. 3c exhibits the images of the P-Fe_2_O_3_-0.25 samples. It was found that the surface of the samples became rough, and a layer of phosphide was distributed on the surface of the Fe_2_O_3_ nanowire arrays. From high magnification SEM images, it could be further found that the as-prepared products possessed an average width of 70 nm (in Fig. 3d). As the phosphorus content was increased, the diameter of the sample increased and the distance between adjacent nanowires became smaller. As shown in Fig. 3e and f, EDS mapping demonstrated that the three elements Fe, P and O were uniformly distributed on the surface of the as-prepared P-Fe_2_O_3_-0.65 nanowire arrays.

**Fig. 4:**
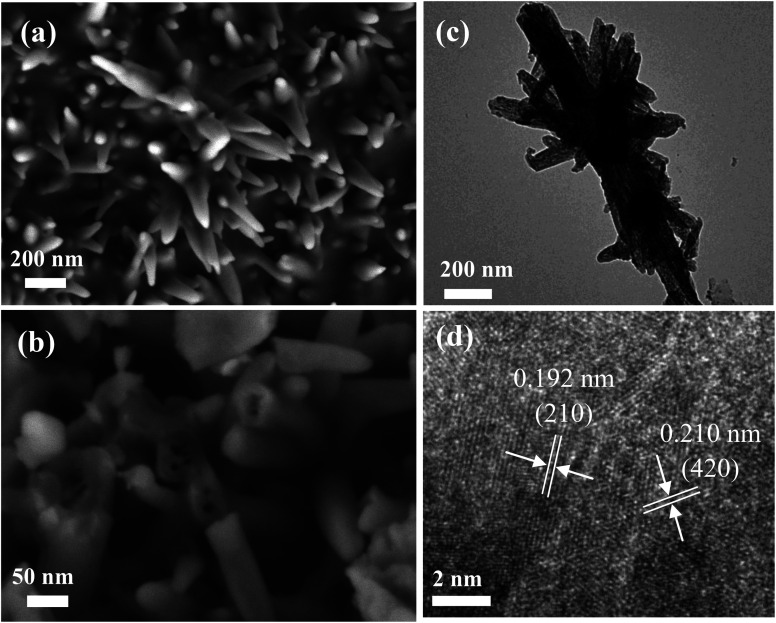
Morphology characteristic of the as-prepared P-Fe_2_O_3_-0.45 nanowire arrays (a and b) SEM images (c) low-magnification TEM images (d) HRTEM images.

Fig. 4a exhibits the TEM image of the P-Fe_2_O_3_ nanowire arrays, and it can be clearly seen that adjacent nanowire arrays were connected with each other to form a stable structure, which benefited in improving the electrocatalytic performance. The high magnification SEM image is presented in Fig. 4b. It was found that the as-prepared sample showed a hollow structure. It contributes to speed up the transmission of electrons. From the high magnification TEM image (Fig. 4c), we can further confirm the uniform distribution of the phosphide layer. HRTEM (Fig. 4d) demonstrated that the measured lattice fringes of 0.192 and 0.210 nm could be indexed to the (210) plane of Fe_2_P and (420) plane of Fe_2_O_3_ samples.

**Fig. 5:**
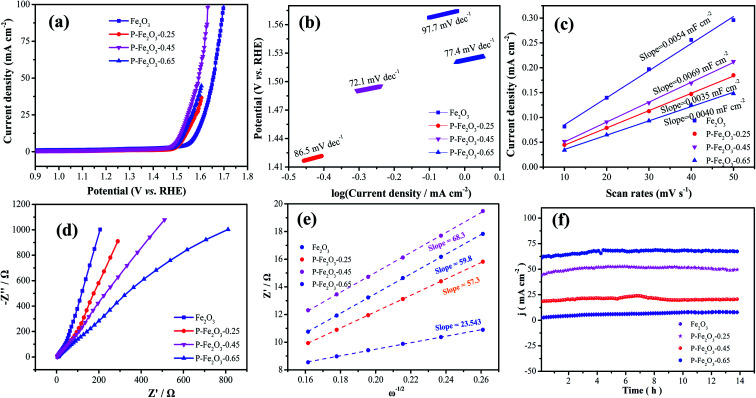
(a) OER performances (a) polarization curves at a scan rate of 2 mV s^−1^ (b) Tafel plots (c) CV curves of double-layer capacitance (d) Nyquist plots (e) *Z*′ as a function of *ω*^−1/2^ plot in low frequency (f) chronoamperometric stability measurements.

To evaluate the OER performance of the as-fabricated products, LSV curves of all electrocatalysts were measured using the standard three-electrode system with 1.0 M KOH solution, as shown in Fig. 5a, with IR compensation at a scan rate of 2 mV s^−1^. It is found that the overpotential of P-Fe_2_O_3_-0.45 nanowire arrays was 270 mV at a current density of 10 mA cm^−2^. Even when the current density was 50 mA cm^−2^, the corresponding overpotential was 330.1 mV, which was lower than that of the Fe_2_O_3_ nanowire arrays (340.7 mV), P-Fe_2_O_3_-0.25 (300.2 mV) and P-Fe_2_O_3_-0.25 (290.4 mV) at 10 mA cm^−2^. It indicated that the weak activity may be due to the fact that with the increase of P content, the overlap of the atomic wave functions of the metal and P decreased, which led to excessive P–H interactions. In addition, P-Fe_2_O_3_-0.45 nanowire arrays possessed a higher current density. To analyze the OER kinetics in Fig. 5b, Tafel slope was obtained through LSV curves. P-Fe_2_O_3_-0.45 nanowire arrays presented a low Tafel slope (72.1 mV dec^−1^), which was smaller than that of the Fe_2_O_3_ nanowire arrays (97.7 mV dec^−1^), P-Fe_2_O_3_-0.25 (86.5 mV dec^−1^) and P-Fe_2_O_3_-0.65 (77.4 mV dec^−1^). P decoration could decrease the overpotential and increase the OER performance. Electrochemical surface area was obtained through electrochemical double-layer capacitance. The *C*_dl_ values of various electrocatalysts are presented in Fig. 5c; it can be seen that P-Fe_2_O_3_-0.45 nanowire arrays showed a higher electrochemical activity, demonstrating that P decoration could improve the active sites. To further study the electron transmission speed of the electrodes, EIS spectra were obtained and are depicted in Fig. 5d. In the high frequency region, the slope of P-Fe_2_O_3_-0.45 nanowire arrays was larger than that of Fe_2_O_3_ nanowire arrays and P-Fe_2_O_3_-0.25 and P-Fe_2_O_3_-0.65, revealing that the samples showed a low ion diffusion resistance.^27^ To further evaluate the charge transport, the low frequency region could be fitted, which could be analyzed through the following equation:

3 *Z* = *R*_s_ + *R*_ct_ + *σ*_w_*ω*^−1/2^

where *σ*_w_ is Warburg factor, *ω* represents angular frequency and *Z* denotes diffusive resistance for OH^−^. From Fig. 5e, it was found that the P-Fe_2_O_3_-0.45 nanowire arrays presented a high slope value as compared to other electrode materials, indicating that the P-Fe_2_O_3_-0.45 samples possessed a fast transmission path for OH^−^. The cycle stability of the as-prepared samples showed that the as-prepared P-Fe_2_O_3_-0.45 nanowire arrays had excellent cycle stability.

**Fig. 6:**
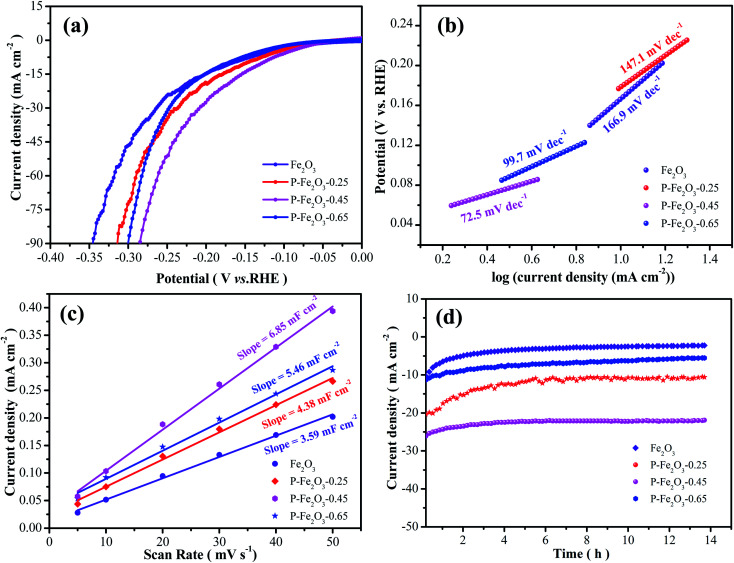
HER performances of the as-obtained samples (a) polarization curves at a scan rate of 5 mV s^−1^ (b) Tafel plots (c) CV curves of double-layer capacitance (*C*_dl_) (d) chronoamperometric stability tests.

To investigate the electrocatalytic performances of the as-obtained samples, HER experiments were conducted in a standard three-electrode system at a scan rate of 5 mV s^−1^. As depicted in Fig. 6a, it was found that the overpotential of P-Fe_2_O_3_-0.45 samples were 126.4 mV at −10 mA cm^−2^, which was lower than that of Fe_2_O_3_ (171.7 mV), P-Fe_2_O_3_-0.25 (149.1 mV) and P-Fe_2_O_3_-0.65 (166.2 mV) electrode materials at −10 mA cm^−2^. The overpotential was significantly reduced, indicating that the P decoration could improve the electrochemical activity.

From Fig. 6b, it was found that the P-Fe_2_O_3_-0.45 nanowire arrays showed a small Tafel slope of 72.5 mV dec^−1^, which was smaller than that of Fe_2_O_3_ (166.9 mV dec^−1^), P-Fe_2_O_3_-0.25 (147.1 mV dec^−1^) and P-Fe_2_O_3_-0.65 (97.2 mV dec^−1^) electrode materials. Electrochemical active surface area was used to estimate the surface activity of the as-obtained samples, as shown in Fig. 6c. It was obviously seen that the P-Fe_2_O_3_-0.45 nanowire arrays (6.85 mF cm^−2^) possessed a higher slope than those electrode materials, such as, P-Fe_2_O_3_-0.65 (5.46 mF cm^−2^), P-Fe_2_O_3_-0.25 (4.38 mF cm^−2^) and P-Fe_2_O_3_ (3.59 mF cm^−2^). The large *C*_dl_ value demonstrated that the as-prepared electrocatalyst presented very active catalytic sites. Long cycle stability was another significant factor for as-prepared electrocatalyst. As shown in Fig. 6d, the as-prepared P-Fe_2_O_3_-0.45 nanowire arrays presented a high stability after 14 h, indicating that the as-prepared electrocatalyst showed excellent catalytic stability.

**Fig. 7:**
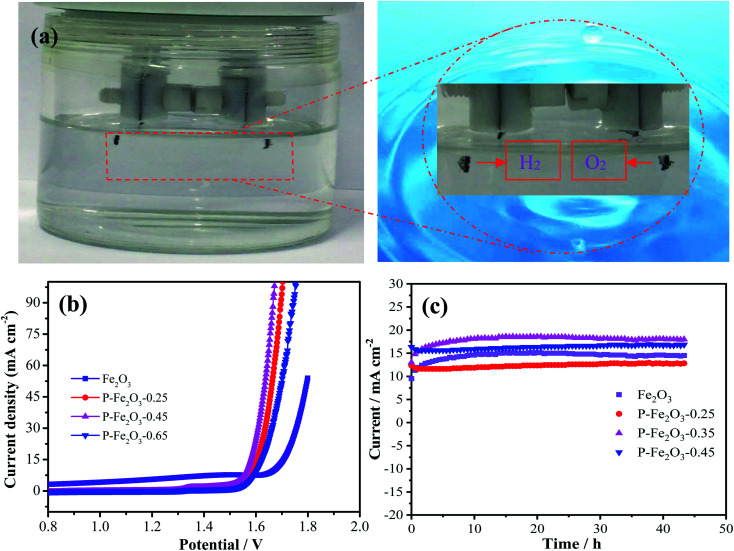
(a) Schematic illustration of overall water splitting (b) polarization curves at a scan rate of 5 mV s^−1^ (c) chronopotentiometry measurements.

Overall water splitting measurement was conducted in an alkaline solution with a two-electrode system by employing P-Fe_2_O_3_-0.45 nanowire arrays as both the anode and the cathode. Carbon cloth was selected as the supporting material due to its low price, good conductivity and excellent flexibility. The corresponding schematic diagram is shown in Fig. 7a. Through the partially enlarged picture, it can be seen that obvious bubbles were generated, and the bubbles could be easily separated, indicating that the prepared electrode material has good application prospects. Fig. 7b shows the LSV curves of the as-obtained samples, and it was found that the P-Fe_2_O_3_-0.45 nanowire arrays showed a low cell voltage of 1.56 V at 10 mA cm^−2^, which was lower than that of P-Fe_2_O_3_-0.25 nanowire arrays (1.59 V), P-Fe_2_O_3_-0.45 nanowire arrays (1.62 V) and Fe_2_O_3_ nanowire arrays (1.67 V). It was further confirmed that the prepared electrode material had a good electrocatalytic performance. Long-term durability of the as-obtained electrocatalyst was also measured. It was found that the current density did not decrease significantly after 43 h. The excellent electrocatalytic performance could be attributed to the following: first, direct growth of Fe_2_O_3_ nanowire arrays on carbon benefits sufficient contact between the electrode and the current collector. Second, phosphating serves as a secondary support, which induces multiple active sites during the electrochemical reaction process, and in turn provides high electrically conductive pathways and accelerates electron transportation speed.^28–30^ In addition, the electrocatalytic performance of the prepared sample was compared with the reported catalyst, and it can be found that the prepared material has good electrocatalytic performance (Table 1).^31–36^

**Table 1:** Comparison of the as-prepared electrocatalysts with reported catalysts

Catalysts	Electrolyte OER/HER	*η* value at 10 mA cm^−2^	Reference
P-Fe_2_O_3_-0.45	1 M KOH (OER)	270 mV	This work
P-Fe_2_O_3_-0.45	1 M KOH (HER)	126.4 mV	This work
NiO/Co_3_O_4_	1 M KOH (OER)	169.5 mV	31
Co-MOF-85	1 M KOH (OER)	203 mV	32
NiCo_2_O_4_@NiMo_2_S_4_	1 M KOH (OER)	159 mV	33
Mo-CoP nanoarrays	1 M KOH (HER)	250 mV	34
NiCoP nanoplates	1 M KOH (HER)	56 mV	35
NiCo-LDH nanosheets	1 M KOH (HER)	100 mV	36

## 4. Conclusion

In summary, we have successfully fabricated novel P-Fe_2_O_3_ nanowire arrays through a simple hydrothermal process. The as-obtained electrode material exhibited excellent electrocatalytic performance, which could be attributed to the P element decoration improving the reaction active sites. The as-obtained P-Fe_2_O_3_-0.45 nanowire arrays exhibited excellent OER activity with a low overpotential of 270 mV at 10 mA cm^−2^ and a low Tafel slope of 72.1 mV dec^−1^. As an HER electrocatalyst, P-Fe_2_O_3_-0.45 nanowire arrays exhibited a low overpotential of 126.4 mV at −10 mA cm^−2^ and a small Tafel slope of 72.5 mV dec^−1^. At the same time, the P-Fe_2_O_3_-0.45 nanowire arrays possessed a low cell voltage of 1.56 V at 10 mA cm^−2^. Thus, P decoration might provide a novel and promising method for designing advanced electrode materials.

## Conflicts of interest

The authors declare no conflict of interest.

## Supplementary Material
